# Together in the Fight against Arthropod-Borne Diseases: A One Health Perspective

**DOI:** 10.3390/ijerph16234876

**Published:** 2019-12-03

**Authors:** Giovanni Benelli, Sengottayan Senthil-Nathan

**Affiliations:** 1 Department of Agriculture, Food and Environment, University of Pisa, via del Borghetto 80, 56124 Pisa, Italy; 2 Division of Biopesticides and Environmental Toxicology, Sri Paramakalyani Centre for Excellence in Environmental Sciences, Manonmaniam Sundaranar University, Alwarkurichi, Tirunelveli 627412, Tamil Nadu, India; senthil@msuniv.ac.in

## 1. Managing Arthropod Vectors and Vector-Borne Diseases: One Health Helps

Arthropod-borne diseases represent a major risk for humans, livestock, pets and wildlife worldwide. The rapid spread of highly aggressive arboviruses and parasites, along with the development of resistance in their arthropod vectors, represent a huge challenge for parasitology, medical entomology and tropical medicine. As highlighted by the Centers for Disease Control and Prevention, to successfully fight arthropod-borne diseases a One Health approach is valuable. Indeed, One Health points out that the human health is strongly connected to the health of animals and the environment. The main aim of One Health is to encourage the cooperation among multiple disciplines to achieve the best health for humans, animals and the environment [1,2].

The present Special Issue includes articles by experts on arthropod vector ecology and control, as well as on the prevention and treatment of arthropod-borne diseases. Herein, special emphasis has been dedicated to three of the main dangerous groups of arthropod-borne diseases: the ones caused by pathogens and/or parasites vectored by mosquitoes (Diptera: Culicidae), tsetse flies (Diptera: Glossinidae) and hard ticks (Ixodida: Ixodidae).

## 2. What Has Been Done

The proper management of mosquito populations is crucial to limit the spread of mosquito-borne diseases, such as malaria, dengue, West Nile, chikungunya, Zika virus and lymphatic filariasis. Therefore, developing novel biocontrol tools is a timely challenge; in particular, entomopathogens may represent a valuable strategy to reduce synthetic pesticide overuse against mosquitoes. Vivekanandhan et al. [3] provided interesting insights on the efficacy of mycelial extracts of the entomopathogen *Beauveria bassiana* (Bals.-Criv.) Vuill. against the filariasis vector *Culex quinquefasciatus* Say. The authors pointed out relevant toxicity of *B. bassiana*-28 ethyl acetate extract on mosquito larvae and pupae, showing that its efficacy was comparable to that of a commercial insecticide based on *B. bassiana*-22. Moreover, the *B*. *bassiana*-28 extract was chemically characterized through GC-MS analyses and its impact on *C*. *quinquefasciatus* in terms of histological damages showed midgut tissue lysis [3].

A further interesting multidisciplinary approach to develop novel and eco-friendly mosquito insecticides is the exploitation of botanical secondary metabolites for pesticide preparation, due to their multiple mechanisms of action making resistance development unlikely [4]. The present Special Issue includes a research [5] focusing on the evaluation of *Acacia nilotica* (L.) Willd. ex Delile (Fabaceae) seed oil and seed pod extract against three important mosquito species: *Anopheles stephensi* Liston, *Aedes aegypti* L. and *Cx*. *quinquefasciatus*. The authors assessed their larvicidal and adulticidal potential, after evaluating the chemical composition of both tested plant-borne products. In particular, about larvicidal effects, they obtained noteworthy LC_50_ values, always lower than 15 mg/L, in full agreement with the criteria recently reported by Pavela [6] and Pavela et al. [7], for the identification of plant essential oils and extracts with highly promising mosquito larvicidal potential.

Still on the advantages of exploiting plants as reservoirs of interesting compounds in the fight against parasites, this Special Issue contains insights on “green” drug development against another dangerous arthropod-borne disease, the human African trypanosomiasis (HAT), caused by *Trypanosoma brucei* parasites, which are vectored by tsetse flies (Diptera: Glossinidae). Ngahang Kamte and coworkers [8] studied the inhibition of *T*. *brucei* TC221 by six essential oils extracted from aromatic plants traditionally used in Cameroon for pharmacological purposes, characterizing the oil chemical composition by GC-MS. Selected major constituents from the essential oils were also tested. The oils from *Azadirachta indica* A. Juss (Meliaceae), *Aframomum daniellii* (Hook. F.) K. Schum. (Zingiberaceae) and *Echinops giganteus* A. Rich. (Asteraceae) achieved low IC_50_ values (i.e., about 10 µg/mL or even lower), as well as selectivity towards mouse embryonic fibroblasts Balb/3T3. Overall, the authors outlined a successful approach to exploit Cameroonian flora as a reservoir for isolating novel products to develop novel and effective HAT drugs [8].

The third highly dangerous group of arthropod-borne diseases considered by this Special Issue is represented by tick-borne diseases (TBDs). Ticks transmit a wide diversity of pathogens causing a number of TBDs [2]. Under the One Health framework, insights on ecology, monitoring and control of hard ticks are welcomed. Herein, Torina et al. [9] proposed a geographical information system (GIS)-based approach for integrated strategies of tick surveillance and control, which is of interest for public health actions. The study was carried out in a natural reserve of Southern Italy (Monte Pellegrino, close to Palermo, in Sicily). Solid findings based on three years of collected data were retrieved, particularly about *Ixodes ventalloi* Gil Collado and *Hyalomma lusitanicum* Koch in two distinct sites; GIS characterized the environmental characteristics of each site, analyzing tick species abundance in relation to time and space [9].

The Special Issue continues with a review by Dente et al. [10] focused on the importance to strengthen arbovirus surveillance in Mediterranean and Black Sea countries within the One Health framework. The authors carefully examined surveillance systems for West Nile, chikungunya, dengue and Rift Valley fever viruses, considering the various human, animal, entomological and environmental sectors involved. The criteria proposed in the conceptual framework developed by the authors to describe integrated surveillance were consistently reported in the context of researches and programs related to integrated surveillance of the above-mentioned arboviral diseases. Notably, these criteria may facilitate the identification and description of operationalized One Health surveillance.

Then, the Special issue ends with a Benchmark article by Brisola Marcondes and Benelli [11], which critically focused on tenuous links among mosquito bites, parasites and pathogens transmitted by these insects, and cancer, stressing that the WHO International Agency for Research on Cancer Monograph Working Group classified *Plasmodium falciparum* infection in holoendemic areas as “probably carcinogenic to humans” (group 2A) [12,13].

## 3. The Future of One Health: Forthcoming Challenges for Entomology

Overall, we are fully aware that the studies published in this Special Issue cannot fully represent the astonishing diversity of the research efforts attempted by scientists working in entomology and parasitology. There is a long and winding road to translate the One Health criteria into practice. On the other hand, we feel that the articles included represent useful examples, boosting the integrated management of three major groups of arthropod vectors of high public health importance.

Besides strengthening One Health research on these important vectors and vector-borne diseases, it is also expected that the present efforts will be useful to promote future multidisciplinary studies on other arthropod parasites and vectors, such as triatomine bugs, sandflies, bed bugs, tabanids, biting midges and botflies (to cite just some examples), which currently cause remarkable medical and veterinary issues, nevertheless suffering from less research on their basic biology, ecology, monitoring and management when compared to mosquitoes and hard ticks.

## References

[1] Dantas-Torres F., Chomel B.B., Otranto D. (2012). Ticks and Tick-borne Diseases: A One Health Perspective. Trends Parasitol..

[2] Benelli G., Duggan M.F. (2018). Management of Arthropod Vector Data–Social and Ecological Dynamics facing the One Health Perspective. Acta Trop..

[3] Vivekanandhan P., Kavitha T., Karthi S., Senthil-Nathan S., Shivakumar M. (2018). Toxicity of *Beauveria bassiana*-28 Mycelial Extracts on Larvae of *Culex quinquefasciatus* Mosquito (Diptera: Culicidae). Int. J. Environ. Res. Public Health.

[4] Jankowska M., Rogalska J., Wyszkowska J., Stankiewicz M. (2018). Molecular Targets for Components of Essential Oils in the Insect Nervous System—A Review. Molecules.

[5] Vivekanandhan P., Venkatesan R., Ramkumar G., Karthi S., Senthil-Nathan S., Shivakumar M. (2018). Comparative Analysis of Major Mosquito Vectors Response to Seed-Derived Essential Oil and Seed Pod-Derived Extract from *Acacia nilotica*. Int. J. Environ. Res. Public Health.

[6] Pavela R. (2015). Essential Oils for the Development of Eco-friendly Mosquito Larvicides: A review. Ind. Crop. Prod..

[7] Pavela R., Maggi F., Iannarelli R., Benelli G. (2019). Plant Extracts for Developing Mosquito Larvicides: From Laboratory to the Field, with Insights on the Modes of Action. Acta Trop..

[8] Ngahang Kamte S., Ranjbarian F., Campagnaro G., Nya P., Mbuntcha H., Woguem V., Womeni H., Ta L., Giordani C., Barboni L. (2017). *Trypanosoma brucei* Inhibition by Essential Oils from Medicinal and Aromatic Plants Traditionally Used in Cameroon (*Azadirachta indica*, *Aframomum melegueta*, *Aframomum daniellii*, *Clausena anisata*, *Dichrostachys cinerea* and *Echinops giganteus*). Int. J. Environ. Res. Public Health.

[9] Torina A., Blanda V., Blanda M., Auteri M., La Russa F., Scimeca S., D’Agostino R., Disclafani R., Villari S., Currò V. (2018). A Geographical Information System Based Approach for Integrated Strategies of Tick Surveillance and Control in the Peri-Urban Natural Reserve of Monte Pellegrino (Palermo, Southern Italy). Int. J. Environ. Res. Public Health.

[10] Dente M., Riccardo F., Nacca G., Ranghiasci A., Escadafal C., Gaayeb L., Jiménez-Clavero M., Manuguerra J., Picard M., Fernández-Pinero J. (2018). Strengthening Preparedness for Arbovirus Infections in Mediterranean and Black Sea Countries: A Conceptual Framework to Assess Integrated Surveillance in the Context of the One Health Strategy. Int. J. Environ. Res. Public Health.

[11] Brisola Marcondes C., Benelli G. (2019). Mosquitoes, infectious diseases and cancer: A connection to study?. Int. J. Environ. Res. Public Health.

[12] Bouvard V., Baan R.A., Grosse Y., Lauby-Secretan B., El Ghissassi F., Benbrahim-Tallaa L. (2012). Carcinogenicity of malaria and of some polyomaviruses. Lancet Oncol..

[13] International Agency for Research on Cancer (2012). A review of human carcinogens: Biological agents. IARC Monographs on the Evaluation of Carcinogenic Risks to Humans.

